# Use of DXA-derived 3D-modeling, as implemented by 3D-Shaper, for the assessment of fracture risk in a population-based setting

**DOI:** 10.1093/jbmr/zjaf120

**Published:** 2025-09-02

**Authors:** Kirsty Huininga, Fjorda Koromani, M Carola Zillikens, Evert F S van Velsen, Fernando Rivadeneira

**Affiliations:** Internal Medicine, Erasmus MC Universitair Medisch Centrum Rotterdam, Rotterdam, 3015GD, The Netherlands; Internal Medicine, Erasmus MC Universitair Medisch Centrum Rotterdam, Rotterdam, 3015GD, The Netherlands; Internal Medicine, Erasmus MC Universitair Medisch Centrum Rotterdam, Rotterdam, 3015GD, The Netherlands; Internal Medicine, Erasmus MC Universitair Medisch Centrum Rotterdam, Rotterdam, 3015GD, The Netherlands; Internal Medicine, Erasmus MC Universitair Medisch Centrum Rotterdam, Rotterdam, 3015GD, The Netherlands

**Keywords:** DXA, 3D-Shaper, Osteoporosis, Bone fragility, Bone Mineral Density (BMD), Fracture risk

## Abstract

Three-dimensional modeling of hip DXA scans has been proposed to partition BMD into cortical surface (csBMD) and trabecular “volumetric” (tvBMD). We assessed in a population-based cohort, whether such partitioning contributes to fracture risk assessment compared to aBMD alone. Participants (*N* = 4908) from the Rotterdam Study comprised 56% women, with a mean age of 67.4 (SD = 10.1) years. Hip DXA scans were analyzed with enCore (GE Lunar) and 3D-Shaper software. Pearson partial correlation was calculated between aBMD, csBMD, and tvBMD at the total hip, femoral neck, and trochanter. Incident fractures were collected from GP or hospital records. During a mean follow-up of 6.8 (SD = 2.5) years, 171 sustained a *hip* fracture, and 1019 *any-type* fractures. Fracture risk estimates were determined as hazard ratios (HR) per SD decrease in BMD, from Cox-regression adjusted for multiple confounders. High correlation (*r* = >.81; *p* < .001) was observed between aBMD and the modeled 3D parameters, and between csBMD and tvBMD (*r* = 0.85; *p* < .001 at the total hip). Lower aBMD was associated with higher incidence of *any-type* (HR = 1.60, 95% CI: 1.43-1.78) and *hip* (HR = 2.46, 95% CI: 1.90-3.17) fracture; lower csBMD with increased risk of *any-type* (HR = 1.44, 95% CI: 1.30-1.60) and *hip* (csBMD HR = 1.76, 95% CI: 1.39-2.23) fracture; and tvBMD with increased risk of *any-type* (HR = 1.62, 95% CI: 1.45-1.80) and *hip* fracture (HR = 2.44, 95% CI: 1.88-3.15). Inclusion of aBMD in models with tvBMD or csBMD resulted in high multicollinearity and unreliable coefficient parameters (VIF > 5). The effect of csBMD was largely attenuated after inclusion of tvBMD in the same model for both *any-type* (csBMD HR = 0.96, 95% CI: 0.81-1.13; tvBMD HR = 1.67, 95% CI: 1.41-1.98) and *hip* (csBMD HR = 0.83, 95% CI: 0.58-1.21; tvBMD HR = 2.82, 95% CI: 1.94-4.10) fractures. Similar results were observed at the neck and intertrochanteric regions. DXA-derived 3D modeled parameters are highly correlated to aBMD and do not provide additional value for the estimation of fracture risk beyond aBMD alone.

## Introduction

Bone fragility, a significant concern in the aging population, is characterized by reduced bone strength and an increased susceptibility to subsequent fractures.^1^ Common sites of fragility fractures are hip, spine, and wrist resulting from reduced bone mass and microstructural degradation without.^2^ The occurrence of fractures in the absence of significant trauma likely indicates underlying bone fragility, necessitating a comprehensive skeletal evaluation.^3^ Osteoporosis is diagnosed via DXA,^4^ measuring areal BMD (aBMD) at the femoral neck and lumbar spine.^5^ A diagnosis of osteoporosis is confirmed with a BMD T-score of −2.5 or lower.^6^ However, using this osteoporosis threshold captures only about 50% of fragility fractures in women.^7^^,^^8^^,^^9^ This may in part be explained by the fact that bone fragility is influenced not just by density but also by a combination of factors, including bone geometry, bone mass distribution, microarchitecture, and the inherent material properties of bone tissue.^10^ Furthermore, aBMD lacks information about the three-dimensional distribution of bone, as trabecular and cortical bone compartments remain indistinguishable in a DXA scan.^11^

Partitioning the contribution of the cortical and trabecular bone compartments from DXA scans has been proposed as a tool for providing clinicians with additional insights about factors which affect bone fragility.^12^ This differentiation is important because cortical and trabecular bone respond differently to mechanical stress and remodeling.^13^^,^^14^ On one hand, cortical bone provides most of the bone’s structural support reaching 40%-90% of the femoral neck’s total strength.^15^ This has been supported by mechanical testing from Johannesdottir et al., who examined over 70 cadaveric femurs and found that cortical bone parameters were strong predictors of femoral strength, especially in femurs with lower trabecular density.^16^ On the other, trabecular bone has a higher surface-to-volume ratio and is more metabolically active. With aging, changes such as cortical thinning and trabecular bone loss occur but cannot be differentiated by BMD measurement alone.^17^ Trabecular bone loss is typically observed in women soon after menopause, presenting with a rapid decline in BMD over 4-8 yr. Whereas, cortical bone loss in both sexes occurs more gradually and persists into older age.^18^^,^^19^ Therefore, novel alternatives for diagnosing bone fragility more accurately are warranted to differentiate BMD change across distinct biological processes.

Computed tomography can provide detailed assessments of bone structure and density,^10^ but its routine clinical use has been hampered by concerns about high radiation exposure and costs.^20^ Recently, HR-pQCT has emerged as an alternative to evaluate bone microarchitecture and its influence on bone strength.^21^ Yet, the applicability of HR-pQCT is limited by the skeletal site assessed, as it predominantly evaluates peripheral bones rather than central sites more susceptible to fragility fractures, such as the hip and spine, confining its use to research settings.

Previous studies have evaluated whether partitioning bone density distribution into cortical and trabecular compartments can enhance fracture prediction.^22–24^ Yet, these studies have been characterized by small sample sizes yielding conflicting results. Recently, 3D-modeling from a DXA projection has been proposed as a method to pattern the bone density distribution of the cortical and trabecular macrostructure separately, in a 3D context.^12^ The introduction of DXA-derived 3D-modeling technology as a method for partitioning volumetric cortical and trabecular bone from hip DXA scans offers encouraging possibilities for gaining deeper understanding of bone properties.^12^

Therefore, we aimed to investigate in a large cohort embedded in the general population if partitioning cortical and trabecular compartments by 3D-modeling of DXA scans contributes to fracture risk assessment compared to using aBMD alone. We assessed the correlation between aBMD, csBMD, and tvBMD parameters at different hip sites (total hip, femoral neck, and trochanter). Then, we estimated the association of csBMD and tvBMD with fracture risk in comparison to aBMD. Subsequently, we examined subgroups or individuals presenting discordant values between aBMD, csBMD, and tvBMD as a means to reveal distinct characteristics that could provide additional insights into bone fragility beyond standard BMD measurements.

## Materials and methods

### Population and study design

**Figure 1:**
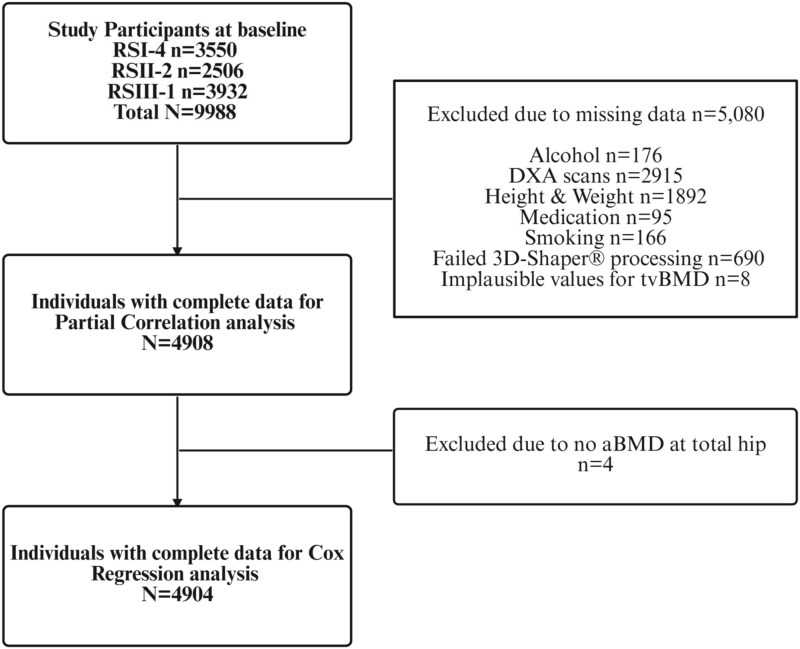
Flowchart of study participants with complete data and exclusions due to missing values. Created by Biorender.com

The Rotterdam study (RS) comprises community dwelling adults aged above 45 yr old. Participants visit the dedicated research center every 3-5 yr and undergo extensive measurements, tests, and interviews.^25^ We included participants (*n* = 4908) from the Rotterdam Study sub-cohorts RSI-4, RSII-2, and RSIII-1 who had undergone DXA measurements between 2002 and 2008 and had complete covariate data (Figure 1). Some participants had missing data for multiple variables (eg, both alcohol consumption and smoking status), while others had missing data for only one variable. A total of eight participants were excluded due to implausible negative tvBMD values. The Rotterdam Study is approved by the Ministry of Health, Welfare, and Sport and the Medical Ethics Committee of Erasmus MC (MEC 02.1015). All participants provided written informed consent.

### Measurements

Areal BMD (aBMD) was measured for all participants using a GE Lunar Prodigy DXA scanner using standardized protocols for scan acquisition. A diagnosis of normal BMD, osteopenia, and osteoporosis at the femoral neck was made according to the WHO definition using the NHANES reference population. All bone density parameters were standardized (mean = 0, SD = 1) and the effect estimates of the regression models are reported per SD decrease.

DXA-derived 3D-modeling parameters were derived via 3D-Shaper software (version 2.7, Galgo Medical) from hip DXA scans. The method uses an algorithm which segments the cortex and computes a 3D map of cortical thickness and density.^12^ The geometry and density distributions were calculated for various regions (including the trochanter, femoral neck, femoral shaft, and total hip) for both cortical and trabecular compartments.^12^ Two parameters were used: tvBMD (mg/cm^3^), representing the density of mineralized tissue within the trabecular bone compartment, and csBMD (mg/cm^2^) computed as the multiplication of the cortical volumetric BMD in mg/cm^3^ and the cortical thickness in cm.^12^ From 7073 DXA scans, we obtained 3D-Shaper measurements for 6383 subjects (90%). During subsequent data checks, eight individuals were found to have negative tvBMD values at the total hip. There values were deemed physiologically implausible, and the corresponding cases were excluded from analysis.

Any-type (excluding toes) and hip fractures were collected from records of general practitioners. Fracture events were identified through computerized systems from GPs within the study region and hospital records. All reported fracture events were independently reviewed and coded by research physicians. Any discrepancies in coding were resolved through a final review conducted by a medical expert.^8^ While fractures of the toes could be systematically excluded, the available records did not distinguish between facial and skull fractures or between hand and finger fractures, so these could not be easily separated. The location of incident fractures was defined as the first fracture occurring at a given location during follow-up. Follow-up time was recorded from baseline (date of the DXA scan) until the occurrence of fracture, death, or the end of the follow-up (January 1, 2013).

Covariates included anthropometric measurements and collection of demographic and lifestyle factors. Age, height, weight, and calculated BMI were assessed at the research center at the time of DXA. Smoking status (current, ex-smoker, or non-smoker) and alcohol consumption (grams of alcohol/per day) were obtained through questionnaires administered during a home visit close to the time of the DXA scan; and information about corticosteroid from pharmacy prescriptions. All individuals included in the study had complete data available for DXA hip scans, fracture history, height, weight, smoking status, alcohol consumption, and corticosteroid use. Individuals with missing information on any of the listed variables were excluded from the analysis.

### Statistical analysis

Descriptive statistics were calculated for all demographic and clinical variables, with means and SD reported for continuous variables and counts and percentages for categorical variables. Pearson correlation coefficients were computed to assess the relationships between aBMD, csBMD, and tvBMD at various hip sites (total hip, femoral neck, and trochanter) and with various demographic and lifestyle factors (such as smoking and alcohol use). These relationships were visualized using scatter plots and correlation matrices. Additionally, partial correlations were performed at the same locations using the covariates sex, age, height, and weight to control for the effect of those factors. Cox proportional hazard models were used to estimate time to fracture for a given aBMD, csBMD, or tvBMD. To test whether tvBMD and csBMD provided additional and independent information for fracture risk estimation compared to aBMD, Cox models containing aBMD, tvBMD, and csBMD were constructed. Four independent models were built to assess these relationships, and the area under the curve (AUC) was calculated to evaluate model performance. To standardize BMD values, individual values were converted to *Z*-scores by subtracting the sample mean and dividing by the sample standard deviation: X – μ/σ, where X represents the individual BMD value, μ is the sample BMD mean, and σ is the sample’s BMD standard deviation. Multivariate models included sex, age, sub-cohort, height, weight, bone density parameters, smoking status (never, ex-smoker, or current), alcohol use (grams/per day), and systemic corticosteroid use (yes/no). To confirm the robustness of our Cox proportional hazards models, we conducted several diagnostic tests. Schoenfeld residuals were used to check the proportional hazards assumptions, where no systematic departures or trends were observed. Residual analyses included plotting martingale residuals over time and evaluating autocorrelation through Ljund–Box test. Furthermore, the normality of residuals was examined using QQ plots and the Shapiro–Wilk test. The linearity assumption was checked by plotting covariates against the log-hazard using Lowess smoothing and performing likelihood ratio tests to compare linear and nonlinear models. To identify subgroups of individuals for which csBMD and tvBMD could potentially provide additional information not captured by DXA we analyzed the data by (1) stratifying the study population in three categories based on aBMD T-score values: normal T-score, osteopenia, and osteoporosis, and (2) examining the subgroup of individuals with discordant values of aBMD, tvBMD, and csBMD. Discordant values were defined as those deviating more than 3SD away from the regression line of either aBMD vs tvBMD, or aBMD vs csBMD, or tvBMD vs csBMD at either the hip, trochanter, or femoral neck. Baseline characteristics of these individuals with discordant measurements were then compared to the overall cohort.

### Post-hoc analysis

All DXA scans of the individuals identified with discordant values of aBMD, tvBMD, and csBMD were visually inspected. This analysis was conducted to explore potential structural or pathological explanations for the observed discordance. During visual inspection of the images, there appeared to be several cases with osteophytes in the hip joint, prompting us to evaluate further information on hip OA. Prevalence of hip OA was then compared between the group of individuals with discordant values of the parameters and the remaining cohort.

## Results

### Basic characteristics

Study participants were on average 67.4 yr old, where 56% were women. Participants had a mean BMI of 27.5 kg/m^2^, aBMD of 0.90 g/cm^2^, tvBMD of 195.9 mg/cm^3^, and csBMD of 124.1 mg/cm^2^ measured at the total hip. The prevalence of osteopenia at the femoral neck was 45% and of osteoporosis 7%; similarly at the total hip the prevalence of osteopenia was 35% and osteoporosis was 6% (Table 1).

**Table 1:** Descriptive characteristics of the study population.

**Characteristics**	**Combined (*n*** = **4908)**	**RSI** **(*n* = 2353)**	**RSII** **(*n* = 921)**	**RSIII** **(*n* = 1634)**
**Sex (*n*, %)**				
**Male**	2171 (44.2)	1047 (44.5)	406 (44.1)	718 (43.9)
**Female**	2737 (55.8)	1306 (55.5)	515 (55.9)	916 (56.1)
**Age,** yr (SD)	67.4 (10.1)	75.1 (5.8)	67.1 (6.2)	56.4 (5.9)
**Mortality (*n*, %)**	881 (18.0)	787 (33.4)	94 (10.2)	0 (0)
**Height,** cm (SD)	168.4 (9.4)	166.8 (9.2)	167.8 (8.8)	171.1 (9.5)
**Weight,** kg (SD)	78.2 (14.0)	76.5 (13.2)	78.7 (13.5)	80.5 (15.0)
**BMI** (SD)	27.5 (4.2)	27.5 (4.1)	27.9 (4.2)	27.4 (4.2)
**Alcohol,** g/p/d (SD)	10.9 (13.1)	11.5 (14.1)	13.3 (15.2)	8.7 (9.6)
**Smoking (*n*, %)**				
**Never**	1500 (30.5)	704 (30.0)	271 (29.4)	525 (32.1)
**Past**	2619 (53.4)	1366 (58.0)	507 (55.1)	746 (45.7)
**Current**	789 (16.1)	283 (12.0)	143 (15.5)	363 (22.2)
**Systemic corticosteroid medication (*N*, %)**				
**Non-user**	4851 (98.8)	2310 (98.2)	910 (98.8)	1631 (99.8)
**User**	57 (1.2)	43 (1.8)	11 (1.2)	3 (0.2)
**Mean BMD T-score (SD)**	−0.97 (1.1)	−1.3 (1.1)	−0.9 (1.0)	−0.6 (1.1)
**BMD *T*-score categories (*n*, %) at femoral neck**				
**Normal**	2375 (48.4)	885 (37.6)	463 (50.2)	1027 (62.9)
**Osteopenia**	2206 (44.9)	1217 (51.7)	415 (45.1)	574 (35.1)
**Osteoporosis**	327 (6.7)	251 (10.7)	43 (4.7)	33 (2.0)
**BMD *T*-score Categories (*n*, %) at total hip *N* = 4905**				
**Normal**	2889 (58.9)	1095 (46.6)	576 (62.5)	1218 (74.5)
**Osteopenia**	1707 (34.8)	1007 (42.9)	308 (33.5)	392 (24.0)
**Osteoporosis**	309 (6.3)	248 (10.5)	37 (4.0)	24 (1.5)
**aBMD, g/cm^2^ (SD)**	0.95 (0.16)	0.91 (0.16)	0.96 (0.15)	1.00 (0.14)
**csBMD, mg/cm^2^ (SD)**	161.6 (25.6)	156.5 (26.3)	163.8 (24.1)	167.7 (23.8)
**tvBMD, mg/cm^3^ (SD)**	157.7 (41.4)	146.6 (40.5)	160.8 (39.0)	172.2 (39.2)
**Fractures (*n*, %)**				
**Hip fracture**	171 (3.4)	154 (6.5)	14 (1.5)	3 (0.2)
**Hip fracture Fup time, yr (SD)**	7.2 (3.3)	8.0(2.6)	7.9(1.4)	5.5(0.9)
**Any-type fractures**	1019(20.8)	756 (32.1)	156 (16.9)	107 (6.5)
**Any-type Fup time, yr (SD)**	6.8 (2.5)	7.6 (2.8)	7.5 (1.9)	5.3 (1.2)
**Crude incidence rate (per 1000 yr)**				
**Mortality**	24.85	41.05	12.9	0
**Hip fractures**	4.87	8.15	1.93	0.33
**Any-type fractures**	30.41	42.3	22.47	12.29

Abbreviations: aBMD, areal bone mineral density; csBMD, cortical surface bone mineral density; Fup, follow-up; tvBMD, trabecular volumetric bone mineral density.

During a mean follow-up time of 6.8 yr (SD = 2.5), 171 participants (3.4%) sustained a hip fracture, and 1019 (20.8%) sustained *any-type* fracture. Incidence rates (per 1000 person-years) were 4.87 for hip fractures, 30.41 for *any-type* fractures, and 24.85 for mortality in the overall group (Table 1). Details on the types of fractures observed and their incidence are provided in Table S1.

### Correlation between aBMD and DXA-derived 3D-modeling parameters

**Figure 2:**
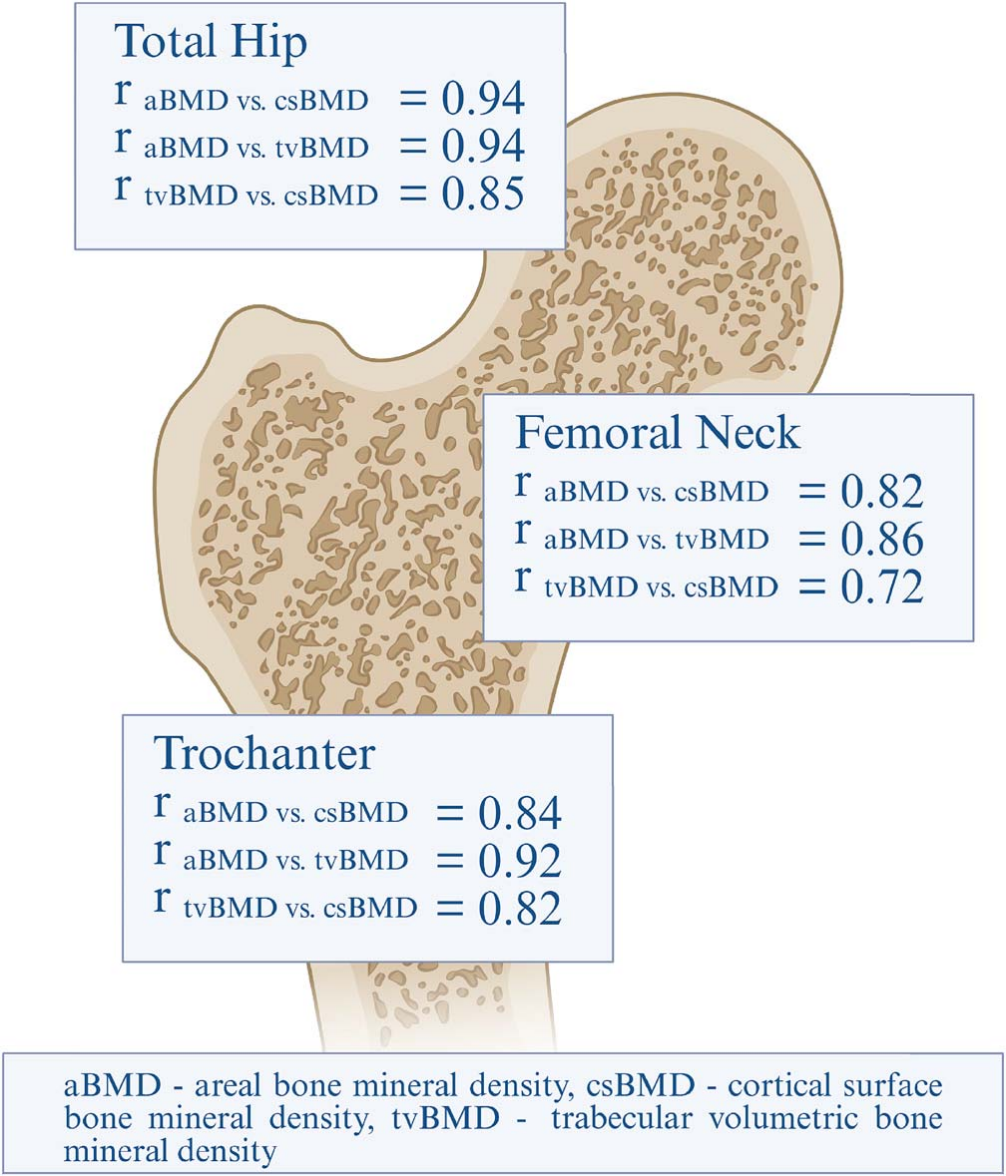
Partial correlations at different locations at the total hip adjusted for sex, age, height, weight, and sub-cohort (*N* = 4908). All *p*-values <.05. Created by Biorender.com

The partial correlations (Figure 2) between aBMD and DXA-derived 3D-modeling parameters were all high (csBMD: *r* = 0.82-0.94; tvBMD: *r* = 0.86-0.94, all *p*-values were <.001) at the total hip, femoral neck, and trochanter. Correlations between csBMD and tvBMD were also strong (*r* = 0.72-0.85, all *p*-values <.001). Sex-specific correlation coefficients are provided in Table S2. The correlations between BMD measurements (aBMD, csBMD, and tvBMD) were similarly high in both sexes across all sites, with slightly higher values were observed in females for some comparisons.

Bivariate correlations with other covariates showed that at the total hip, all BMD measurements had moderate negative correlations with age (aBMD: *r* = −0.34, csBMD: *r* = −0.27, tvBMD: *r* = −0.35, all *p*-values <.001) and female sex (aBMD: *r* = −0.35, csBMD: *r* = −0.32, and tvBMD: *r* = −0.21, all *p*-values <.001); and positive correlations with height (aBMD: *r* = 0.37, csBMD: *r* = 0.36, and tvBMD: *r* = 0.21, all *p*-values <.001), and weight (aBMD: *r* = 0.47, csBMD: *r* = 0.51, and tvBMD: *r* = 0.38, all *p*-values <.001). Similar results were observed at the trochanter and femoral neck regions (Table S3).

### Risk for any-type and hip fractures

After adjusting for cohort, age, sex, height, weight, smoking status, systemic corticosteroid, and alcohol use, aBMD was associated with an increased risk for any-type (HR = 1.60, 95% CI: 1.43-1.78) and hip fracture (HR = 2.46, 95% CI: 1.90-3.17). Similarly, lower tvBMD was associated with higher risk for any-type (HR = 1.62, 95% CI: 1.45-1.80), and hip fractures (HR = 2.44, 95% CI: 1.88-3.15), while lower csBMD with increased risk for any-type (HR = 1.44, 95% CI: 1.30-1.60), and hip fractures (HR = 1.76, 95% CI: 1.39-2.23). Inclusion of tvBMD or csBMD in the same regression model with aBMD gave rise to high inflation (VIF = 9.45) due to high multicollinearity and resulted in unreliable risk coefficient parameters. Inclusion of both tvBMD and csBMD in the same model (VIF > 5), resulted in csBMD not associated with *any-type* fractures (HR = 0.96, 95% CI: 0.81-1.13), while tvBMD remained significantly correlated with increased *any-type* fracture risk (HR = 1.67, 95% CI: 1.41-1.98). Similar results were observed for hip fractures, where tvBMD was significantly associated (HR = 2.82, 95% CI: 1.94-4.10), while csBMD was not (HR: 0.83, 95% CI: 0.58-1.21). Furthermore, risk estimates at both the trochanter and femoral neck regions were very similar (Table S4 and S5). No substantial differences in risk estimates were observed after stratification into WHO-defined BMD *T*-score categories (Table 2).

**Table 2:** The relationship between BMD measurements with DXA or DXA-derived 3D-modeling parameters and incident fractures at the total hip. Results are shown both in the combined population and stratified by BMD *T*-score categories.

**Cox proportional hazards model for outcome any-type fracture (HR (95% CI))**				
	**Combined** *N* = 4904 Events = 603	**Normal** *N* = 2375 Events = 178	**Osteopenia** *N* = 2202 Events = 352	**Osteoporosis** *N* = 327 Events = 73
**Model 1**	1.60 (1.43-1.78)	1.62 (1.26-2.07)	1.49 (1.18-1.87)	1.67 (0.97-2.91)
**Model 2**	1.44 (1.30-1.60)	1.47 (1.18-1.84)	1.13 (0.92-1.38)	1.37 (0.81-2.31)
**Model 3**	1.62 (1.45-1.80)	1.53 (1.23-1.90)	1.61 (1.31-1.97)	1.51 (0.89-2.55)
**Model 4**	zcsBMD 0.96 (0.81-1.13) ztvBMD 1.67 (1.41-1.98)	zcsBMD 1.15 (0.84-1.59) ztvBMD 1.38 (1.02-1.88)	zcsBMD 0.82 (0.65-1.04) ztvBMD 1.79 (1.40-2.30)	zcsBMD 1.28 (0.75-2.20) ztvBMD 1.50 (0.84-2.66)
**Cox proportional hazards model for outcome hip fracture (HR (95% CI))**				
	**Combined** (*n* = 4904, Events = 130)	**Normal** (*n* = 2375, Events = 18)	**Osteopenia** (*n* = 2202, Events = 80)	**Osteoporosis** (*n* = 327, Events = 32)
**Model 1**	2.46 (1.90-3.17)	1.57 (0.72-3.46)	2.41 (1.50-4.01)	2.57 (1.20-5.49)
**Model 2**	1.76 (1.39-2.23)	1.10 (0.57-2.12)	1.57 (1.03-2.41)	0.70(0.35-1.42)
**Model 3**	2.44 (1.88-3.15)	1.51 (0.76-3.00)	2.83 (1.81-4.42)	1.38 (0.65-2.94)
**Model 4**	zcsBMD: 0.83 (0.58-1.21) ztvBMD: 2.82 (1.94-4.10)	zcsBMD 0.68 (0.26-1.79) ztvBMD: 1.97 (0.77-5.06)	zcsBMD: 0.88 (0.54-1.43) ztvBMD: 3.05 (1.80-5.15	zcsBMD: 0.53 (0.24-1.17) ztvBMD: 1.79(0.91-3.95)

Model 1: Cohort + Age + Sex + Height + Weight + Smoking status + Systemic corticosteroid use + Alcohol use + zaBMD.

Model 2: Cohort + Age + Sex + Height + Weight + Smoking status + Systemic corticosteroid use + Alcohol use + zcsBMD.

Model 3: Cohort + Age + Sex + Height + Weight + Smoking status + Systemic corticosteroid use + Alcohol use + ztvBMD.

Model 4: Cohort + Age + Sex + Height + Weight + Smoking status + Systemic corticosteroid use + Alcohol use + zcsBMD + ztvBMD.

Abbreviations: zaBMD, *Z*-score areal bone mineral density; zcsBMD, *Z*-score cortical surface bone mineral density; ztvBMD, *Z*-score trabecular volumetric bone mineral density.

When assessing the predictive performance of models with and without DXA-derived 3D-modeling parameters, we found that: for *any-type* fractures, the AUC for the model with aBMD alone was 69.4 (95% CI: 0.66-0.70), the model with csBMD alone AUC = 68.2 (95% CI: 0.65-0.70) and the model with tvBMD alone AUC = 69.5 (95% CI: 0.66-0.71). Including of both tvBMD and csBMD in the model did not improve significantly the AUC compared to the model with aBMD alone AUC = 69.5 (95% CI: 0.66-0.71). Similarly, in models for hip fracture with and without 3D DXA parameters, the AUC for the model containing aBMD was 63.8 (95% CI: 0.66-0.70); being very similar to the ones of csBMD (AUC = 62.3, 95% CI: 0.64-0.69) and tvBMD (AUC = 64.0, 95% CI: 0.66-0.71) alone. Inclusion of both tvBMD and csBMD in the model did not improve the AUC compared to the model with aBMD alone (AUC = 64.1, 95% CI: 0.66-0.71) (Table 3).

**Table 3:** Time dependent AUC values at 5 yr for both any-type and hip fractures.

**Any-type fractures**	**AUC (95% CI)**
**aBMD**	69.4 (0.67-0.71)
**csBMD**	68.2 (0.66-0.70)
**tvBMD**	69.5 (0.67-0.71)
**csBMD + tvBMD**	69.5 (0.67-0.71
**Hip fractures**	
**aBMD**	63.8 (0.67-0.71)
**csBMD**	62.3 (0.65-0.70)
**tvBMD**	64.0 (0.67-0.71)
**csBMD + tvBMD**	64.1 (0.67-0.71)

All AUC values reported here were adjusted for Cohort + Age + Sex + Height + Weight + Smoking status + Systemic corticosteroid use + Alcohol use + the bone parameter mentioned in the table.

### Discordant values of aBMD, tvBMD, and csBMD

In total, we identified 76 individuals with discordant values of aBMD, tvBMD, or csBMD, of which 53 at the femoral neck, 47 at the trochanter, and 35 individuals at the total hip. The distribution of discordant values across aBMD, tvBMD, and csBMD is shown in Figure S1. Individuals with discordant values of aBMD, tvBMD, or csBMD at the total hip were significantly younger and consumed less alcohol compared to the overall cohort, but the latter difference was not statistically significant after adjusting for age and sex (Table S6). While OA was suspected based on visual inspection of the scans due to presence of osteophytes, participants with discordant values of aBMD, tvBMD, or csBMD did not have a significant higher prevalence of OA at the hip. No other differences were observed.

## Discussion

This study aimed to determine whether the claimed partitioning of BMD into cortical and trabecular compartments using 3D-modeling of DXA scans (as estimated by 3D-Shaper), enhances fracture risk assessment compared to the standard aBMD measurement. Our findings showed that while DXA-derived 3D-modeling parameters were significantly associated with *any-type* and hip fracture risk, they did not show added predictive ability for fracture risk over aBMD measured at the femoral neck, trochanter, or total hip. In our large cohort embedded in the general population, we observed that both csBMD and tvBMD measurements were strongly correlated with aBMD, and assessing their combined use in risk prediction models was not feasible due to high multicollinearity. Furthermore, when both csBMD and tvBMD were included in the models, only tvBMD was significantly associated with fracture risk. Findings were similar across all hip locations (femoral neck, trochanter, and total hip).

Further analysis in individuals stratified by WHO-defined *T*-score groups (normal BMD, osteopenia, and osteoporosis), showed no differences across strata. This finding challenges the precept that the clinical utility of 3D-modeled parameters could be confined to a particular group of patients (ie, those with low BMD). To evaluate whether other potential groups of individuals could benefit from partitioning cortical and trabecular BMD, we further investigated individuals presenting discordant estimates of tvBMD or csBMD in relation to aBMD, either at the femoral neck, trochanter, or total hip regions. We identified 76 individuals with discordant values of aBMD, csBMD, and tvBMD, but we observed no differences in population characteristics that could explain the observed discrepancies or that point to potential applications in special groups of patients. Rather, discrepant measurements may be more likely a consequence of (positioning) artifacts affecting the 3D-modeling.

Demonstrating that the DXA-derived 3D-modeling parameters can add to fracture risk prediction independently of aBMD requires overcoming the limitation set by the very high correlation of these DXA-derived 3D-modeling parameters with aBMD and between them. For instance, we found a very strong correlation between aBMD, tvBMD, and csBMD at the total hip with rho ranging from 0.82-0.94. A study examining these parameters in a subset of participants of the ACTIVE trial (*N* = 500)^26^ also reported strong correlations at the total hip between aBMD and csBMD (*r* = 0.70) as well as between aBMD and tvBMD (*r* = 0.84), albeit somewhat lower than the correlation estimates we report. Correlation between parameters can be influenced by outliers, degrees of variance in the data, and magnitude level of the measurement. However, standard deviations of these BMD parameters were not reported, making direct comparisons challenging. Our study is based on the general population, whereas the referenced study is about ten times smaller and based on trial participants with severe osteoporosis (who were eligible for anabolic therapy with abaloparatide and teriparatide). The larger sample size and broader range of BMD values in our study theoretically provide more robust and generalizable estimates of the actual correlation between these parameters. Altogether, DXA-derived 3D modeling measurements are strongly correlated with aBMD, limiting them from providing substantial additional clinical value in fracture risk prediction.

Previous studies employing DXA-derived 3D-modeling have proposed that the csBMD and tvBMD parameters enhance hip fracture risk prediction beyond traditional aBMD at different hip locations; particularly after stratifying for normal, osteopenia, and osteoporosis *T*-score categories.^27^^,^^28^ We did not replicate such findings. A population-based study on Japanese women (*n* = 1331) examined the potential of DXA-derived 3D-modeling to predict hip fractures and found that tvBMD improved risk prediction over aBMD measured at the femoral neck, but not at the total hip.^27^ Yet, correlations between aBMD and DXA-derived 3D-modeling parameters or tests of multicollinearity were not reported in the study. As a result, it remains uncertain whether similar issues with multicollinearity were encountered, or how potential high parameter correlation might have influenced the reliability of their model estimates. Similarly, a case-control study assessing DXA-derived 3D-modeling parameters in postmenopausal women with hip fractures^28^ found that in cases tvBMD was more markedly reduced than csBMD at the femoral neck and total hip. Yet, DXA-derived 3D-modeling parameters had similar predictive performance as aBMD at the total hip and femoral neck. To date, no other large studies have examined whether DXA-derived 3D-modeling parameters provide additional information beyond aBMD.

The rationale behind seeking improvement of fracture risk prediction through statistical shape and density modeling of the proximal femur is valid. A previous study in elderly men demonstrated that such 3D-modeling of bone geometry and density distribution could add to fracture prediction beyond aBMD when applied to QCT imaging.^29^ Yet, DXA-derived 3D-modeling has technical limitations in how these parameters are derived in a 2D imaging plane. Specific challenges were already pointed out in the ex vivo study set to validate the DXA-derived 3D-modeling approach against QCT.^30^ High correlation of the density distributions between the DXA-derived 3D-modeling parameters and with the QCT scans can be explained by correlation with overall density. The ex-vivo study also highlighted that while DXA-derived 3D-modeling parameters can reconstruct the actual bone geometry and estimate the corresponding strength, their predictive value is similar to that of traditional aBMD, already suggesting limited incremental benefit in fracture prediction. Additionally, the cortical measures from the DXA-derived 3D-modeling are obtained using a statistical model built from principal components of variation in shape and density distribution that is projected onto the DXA image. A concern previously highlighted by Whitmarsh et al.,^31^ is that the model lacks a parameter that describes the cortical thickness or cortical density. This could lead to inflated or inaccurate estimates of cortical thickness and density, potentially impacting the reliability of DXA-derived 3D-modeling predictive value for fractures. Furthermore, errors in femur positioning during DXA acquisition may introduce additional variability in 3D derived 3D-modeling estimates, leading to misleading results further limiting its clinical applicability.^31–33^ Our null findings align with these technical concerns, suggesting that DXA-derived 3D-modeling tvBMD and csBMD provide similar information as traditional aBMD.

This study is not free of limitations. While participants are set to be a representative sample of the general population, the study is prone to healthy volunteer selection bias. However, stratifying by osteoporosis or osteopenia diagnosis did not show different level of correlation or fracture prediction ability across strata. Furthermore, we did not have data to differentiate between the locations of hip fractures (eg, femoral head, femoral neck, intertrochanter) and how they were caused (fall, high-velocity event, or blow to the hip). Therefore, we may have overlooked potential variations in bone density and structural integrity that may be unique to each fracture type. Nevertheless, although the exact mechanisms of fracture remain unknown, this is unlikely to have influenced the overall conclusions. Another limitation of our study is the inability to evaluate DXA-derived 3D-modeling parameters at the lumbar spine, as the technology does not currently support this feature. The lumbar spine is an important site of osteoporotic fracture and predominately consists of trabecular bone, which may respond differently to mechanical stress and metabolic changes compared to the hip. One limitation of our analysis is that the Cox proportional hazards model did not account for competing risks, particularly death, which could have influenced the estimation of hazard ratios by treating death as a censored event. However, this limitation would affect BMD estimations using both DXA and DXA-derived 3D-modeling parameters similarly.

In conclusion, DXA-derived 3D-modeling parameters (as estimated by 3D-Shaper), including csBMD and tvBMD were highly correlated with aBMD across hip sites. Notably, csBMD did not predict *any-type* and hip fracture risk when analyzed together with tvBMD, contrasting with prior studies that emphasize cortical bone’s role in fracture susceptibility. None of the DXA-derived 3D-modeling parameters provided additional value for fracture risk prediction beyond that of aBMD alone.

## Supplementary Material

R1_Supplementary_Figure_1_zjaf120

R1_Supplementary_Table_1_zjaf120

R1_Supplementary_Table_2_zjaf120

R1_Supplementary_Table_3_zjaf120

R1_Supplementary_Table_4_zjaf120

R1_Supplementary_Table_5_zjaf120

R1_Supplementary_Table_6_zjaf1

## Data Availability

Data can be obtained upon request. Requests should be directed towards the management team of the Rotterdam Study (datamanagement.ergo@erasmusmc.nl), which has a protocol for approving data requests. Because of restrictions based on privacy regulations and informed consent of the participants, data cannot be made freely available in a public repository. The Rotterdam Study has been approved by the Medical Ethics Committee of the Erasmus MC (registration number MEC 02.1015) and by the Dutch Ministry of Health, Welfare and Sport (Population Screening Act WBO, license number 1071272-159521-PG). The Rotterdam Study has been entered into the Netherlands National Trial Register (NTR; www.trialregister.nl) and into the WHO International Clinical Trials Registry Platform (ICTRP; https://apps.who.int/trialsearch/) under shared catalogue number NTR6831. All participants provided written informed consent to participate in the study and to have their information obtained from treating physicians.
