# Supplementary figures and images for: Use of DXA-derived 3D-modeling, as implemented by 3D-Shaper, for the assessment of fracture risk in a population-based setting
Source: J Bone Miner Res. 2025 Sep 2;41(2):128–35. doi: 10.1093/jbmr/zjaf120 (PMC12865847; doi:10.1093/jbmr/zjaf120)

## Slide 1
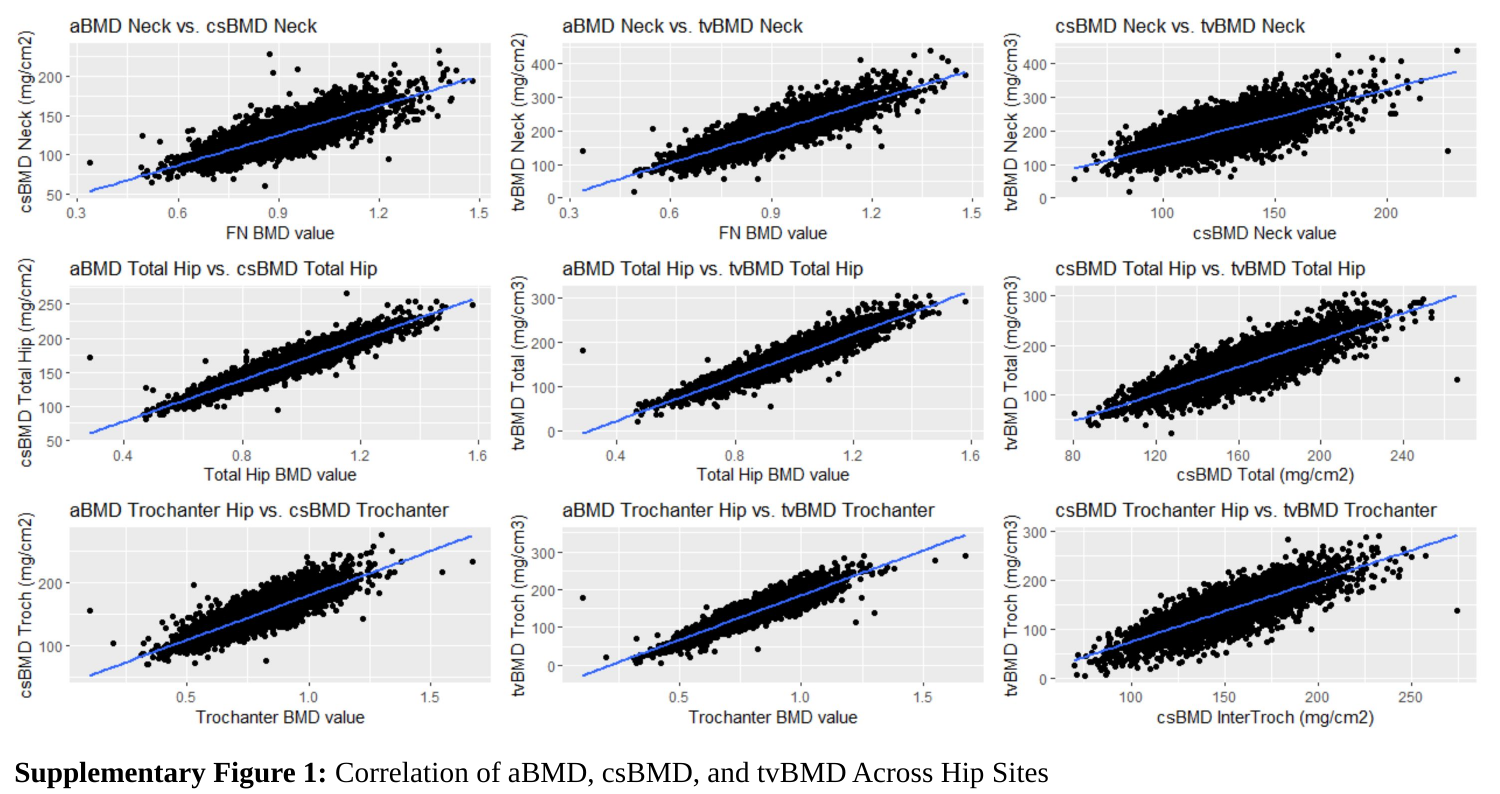

Supplementary Figure 1: Correlation of aBMD, csBMD, and tvBMD Across Hip Sites

Supplement: R1_Supplementary_Figure_1_zjaf120 [file r1_supplementary_figure_1_zjaf120.pptx]
