# Supplementary material for: Use of DXA-derived 3D-modeling, as implemented by 3D-Shaper, for the assessment of fracture risk in a population-based setting
Source: J Bone Miner Res. 2025 Sep 2;41(2):128–35. doi: 10.1093/jbmr/zjaf120 (PMC12865847; doi:10.1093/jbmr/zjaf120)
Supplement: R1_Supplementary_Table_1_zjaf120 [file r1_supplementary_table_1_zjaf120.docx]

**Supplementary Table 1.** Observed fractures and their incidence in the study population

| **Fracture Type (n, %)** | **Combined (n=4908)** | **RSI**  **(n=2353)** | **RSII**  **(n=921)** | **RSIII**  **(n=1634)** |
| --- | --- | --- | --- | --- |
| Skull | 42 (0.9) | 29 (1.2) | 6 (0.7) | 7 (0.4) |
| Vertebral | 280 (5.7) | 213 (9.1) | 52 (5.6) | 15 (0.9) |
| Humerus | 115 (2.3) | 96 (4.1) | 10 (1.1) | 9 (0.6) |
| Rib | 80 (1.6) | 56 (2.3) | 10 (1.1) | 14 (0.9) |
| Sternum | 4 (0.1) | 3 (0.1) | 1 (0.1) | 0 (0.0) |
| Hand | 150 (3.1) | 101 (4.3) | 23 (2.4) | 26 (1.6) |
| Foot | 87 (1.8) | 42 (1.8) | 21 (2.3) | 24 (1.5) |
| Metatarsal | 77 (1.6) | 54 (2.2) | 13 (1.4) | 10 (0.6) |
| Upper arm | 59 (1.2) | 46 (2.0) | 5 (0.05) | 8 (0.5) |
| Lower arm | 68 (1.4) | 47 (2.0) | 12 (1.3) | 9 (0.6) |
| Femur | 13 (0.26) | 10 (0.4) | 3 (0.3) | 0* |
| Ankle | 50 (1.0) | 45 (1.9) | 1 (0.1) | 4 (0.2) |
| Lower leg | 97 (2.0) | 61 (2.6) | 23 (2.5) | 13 (0.8) |
| Hip | 171 (3.5) | 154 (6.5) | 14 (1.5) | 3 (0.2) |
| Wrist | 277 (5.6) | 215 (9.1) | 34 (3.7) | 28 (1.7) |
| Pelvis | 55 (1.1) | 45 (1.9) | 7 (0.8) | 3 (0.2) |
| Any-type* | 1019 (20.8) | 756 (32) | 156 (16.9) | 107 (6.5) |
| *Any-type of fracture refers to the first fracture an individual experienced in any location. If fractures in multiple locations occurred on the same day, or if additional fractures occurred in the same location after the first one had already been recorded, these were not counted as new. Each individual contributed only once in the any-type fracture category  ** Coding changed in RSIII cohort and this variable was no longer collected | | | | |
