# Supplementary material for: Use of DXA-derived 3D-modeling, as implemented by 3D-Shaper, for the assessment of fracture risk in a population-based setting
Source: J Bone Miner Res. 2025 Sep 2;41(2):128–35. doi: 10.1093/jbmr/zjaf120 (PMC12865847; doi:10.1093/jbmr/zjaf120)
Supplement: R1_Supplementary_Table_2_zjaf120 [file r1_supplementary_table_2_zjaf120.docx]

**Supplementary Table 2.** Sex-specific correlations for male and female at the femoral neck, trochanter and the total hip. All p-values are below <0.05.

|  | **Femoral Neck** | | | **Trochanter** | | | **Total Hip** | | |
| --- | --- | --- | --- | --- | --- | --- | --- | --- | --- |
|  | **aBMD vs. csBMD** | **aBMD vs. tvBMD** | **csBMD vs. tvBMD** | **aBMD vs. csBMD** | **aBMD vs. tvBMD** | **csBMD vs. tvBMD** | **aBMD vs. csBMD** | **aBMD vs. tvBMD** | **csBMD vs. tvBMD** |
| **Male** | 0.78 | 0.89 | 0.66 | 0.83 | 0.93 | 0.78 | 0.92 | 0.93 | 0.81 |
| **Female** | 0.86 | 0.89 | 0.76 | 0.85 | 0.94 | 0.79 | 0.95 | 0.95 | 0.87 |
