# Supplementary material for: Use of DXA-derived 3D-modeling, as implemented by 3D-Shaper, for the assessment of fracture risk in a population-based setting
Source: J Bone Miner Res. 2025 Sep 2;41(2):128–35. doi: 10.1093/jbmr/zjaf120 (PMC12865847; doi:10.1093/jbmr/zjaf120)
Supplement: R1_Supplementary_Table_3_zjaf120 [file r1_supplementary_table_3_zjaf120.docx]

**Supplementary Table 3.** Correlations between bone parameters and demographic factors, unadjusted for other covariates

| **Correlation** | **aBMD** | **P-value** | **csBMD** | **P-value** | **tvBMD** | **P-value** |
| --- | --- | --- | --- | --- | --- | --- |
| **Total Hip** | | | | | | |
| Age | -0.34 | >0.001 | -0.27 | >0.001 | -0.35 | >0.001 |
| Sex | -0.35 | >0.001 | -0.32 | >0.001 | -0.21 | >0.001 |
| Height | 0.37 | >0.001 | 0.36 | >0.001 | 0.21 | >0.001 |
| Weight | 0.47 | >0.001 | 0.51 | >0.001 | 0.38 | >0.001 |
| **Femoral Neck** | | | | | | |
| Age | -0.34 | >0.001 | -0.23 | >0.001 | -0.38 | >0.001 |
| Sex | -0.30 | >0.001 | -0.14 | >0.001 | -0.04 | 0.04 |
| Height | 0.38 | >0.001 | 0.23 | >0.001 | 0.10 | >0.001 |
| Weight | 0.42 | >0.001 | 0.27 | >0.001 | 0.24 | >0.001 |
| **Trochanter** | | | | | | |
| Age | -0.28 | >0.001 | -0.25 | >0.001 | -0.30 | >0.001 |
| Sex | -0.46 | >0.001 | -0.26 | >0.001 | -0.26 | >0.001 |
| Height | 0.44 | >0.001 | 0.30 | >0.001 | 0.25 | >0.001 |
| Weight | 0.51 | >0.001 | 0.42 | >0.001 | 0.40 | >0.001 |
| aBMD – areal bone mineral density, csBMD – Cortical surface bone mineral density, tvBMD – Trabecular volumetric bone mineral density | | | | | | |
