# Supplementary material for: Use of DXA-derived 3D-modeling, as implemented by 3D-Shaper, for the assessment of fracture risk in a population-based setting
Source: J Bone Miner Res. 2025 Sep 2;41(2):128–35. doi: 10.1093/jbmr/zjaf120 (PMC12865847; doi:10.1093/jbmr/zjaf120)
Supplement: R1_Supplementary_Table_4_zjaf120 [file r1_supplementary_table_4_zjaf120.docx]

**Supplementary Table 4.** The relationship between BMD measured with DXA or DXA-derived 3D-modelling parameters and incident fractures at the trochanter. Results are shown both in the combined population and stratified by BMD T-score categories

| **Cox Proportional Hazards Model for outcome any-type fracture (HR (95% CI))** | | | | |
| --- | --- | --- | --- | --- |
|  | **Combined**  N=4904  Events= 603 | **Normal**  N=2375  Events= 178 | **Osteopenia**  N=2202  Events 352 | **Osteoporosis**  N=327  Events =73 |
| **Model 1** | 1.58 (1.41-1.76) | 1.56 (1.23-1.97) | 1.34 (1.10-1.62) | 1.68 (1.07-2.64) |
| **Model 2** | 1.41 (1.27-1.55) | 1.43 (1.16-1.75) | 1.06 (0.88-1.29) | 1.61 (0.94-2.75) |
| **Model 3** | 1.53 (1.38-1.67) | 1.49 (1.21-1.83) | 1.39 (1.17-1.66) | 1.34 (0.88-2.03) |
| **Model 4** | zcsBMD  1.06 (0.91-1.22)  ztvBMD  1.47 (1.26-1.70) | zcsBMD  1.19 (0.91-1.55)  ztvBMD  1.34 (1.03-1.74) | zcsBMD  0.84 (0.67-1.05)  ztvBMD  1.51 (1.24-1.85) | zcsBMD  1.46 (0.80-2.67)  ztvBMD  1.17 (0.73-1.87) |
| **Cox Proportional Hazards Model for outcome hip fracture (HR (95% CI))** | | | | |
|  | **Combined** (n=4904, Events = 130) | **Normal**  (n=2375, Events =18) | **Osteopenia**  (n=2202, Events=80) | **Osteoporosis**  (n=327, Events = 32) |
| **Model 1** | 2.26 (1.76-2.91) | 1.44 (0.71-2.92) | 1.84 (1.23-2.78) | 2.18 (1.14-4.16) |
| **Model 2** | 1.75 (1.39-2.19) | 1.17 (0.64-2.14) | 1.47 (0.98-2.21) | 0.87(0.41-1.83) |
| **Model 3** | 1.90 (1.52-2.38) | 1.19 (0.65-2.17) | 1.93 (1.35-2.77) | 1.06 (0.59-1.88) |
| **Model 4** | zcsBMD: 1.18 (0.86-1.64)  ztvBMD: 1.69 (1.22-2.33) | zcsBMD  1.09 (0.48-2.43)  ztvBMD  1.12 (0.5-2.52) | zcsBMD: 0.99 (0.62-1.59) ztvBMD: 1.94 (1.27-2.95) | zcsBMD: 0.80 (0.34-1.85) ztvBMD: 1.14 (0.60-2.19) |
| Model 1: Cohort + Age + Sex + Height + Weight + Smoking Status + Systemic corticosteroid use + Alcohol use + zaBMD  Model 2: Cohort + Age + Sex + Height + Weight + Smoking Status + Systemic corticosteroid use + Alcohol use + zcsBMD  Model 3: Cohort + Age + Sex + Height + Weight + Smoking Status + Systemic corticosteroid use + Alcohol use + ztvBMD  Model 4: Cohort + Age + Sex + Height + Weight + Smoking Status + Systemic corticosteroid use + Alcohol use + zcsBMD + ztvBMD  zaBMD – Z-score areal bone mineral density, zcsBMD – Z-score cortical surface bone mineral density, ztvBMD – Z-score trabecular volumetric bone mineral density | | | | |
