# Supplementary material for: Use of DXA-derived 3D-modeling, as implemented by 3D-Shaper, for the assessment of fracture risk in a population-based setting
Source: J Bone Miner Res. 2025 Sep 2;41(2):128–35. doi: 10.1093/jbmr/zjaf120 (PMC12865847; doi:10.1093/jbmr/zjaf120)
Supplement: R1_Supplementary_Table_5_zjaf120 [file r1_supplementary_table_5_zjaf120.docx]

**Supplementary Table 5.** The relationship between BMD measured with DXA or DXA-derived 3D-modelling parameters and incident fractures at the femoral neck. Results are shown both in the combined population and stratified by BMD T-score categories

| **Cox Proportional Hazards Model for outcome any-type fracture (HR (95% CI))** | | | | |
| --- | --- | --- | --- | --- |
|  | **Combined**  N=4904  Events= 603 | **Normal**  N=2375  Events= 178 | **Osteopenia**  N=2202  Events 352 | **Osteoporosis**  N=327  Events =73 |
| **Model 1** | 1.52 (1.40-1.69) | 1.44 (1.10-1.88) | 1.58 (1.17-2.12) | 1.82 (0.86-3.84) |
| **Model 2** | 1.36 (1.24-1.49) | 1.29 (1.07-1.55) | 1.08 (0.90-1.30) | 1.41 (0.81-2.46) |
| **Model 3** | 1.60 (1.45-1.78) | 1.55 (1.24-1.95) | 1.68 (1.36-2.09) | 1.66 (0.97-2.83) |
| **Model 4** | zcsBMD  1.03 (0.92-1.17)  ztvBMD  1.57 (1.37-1.79) | zcsBMD  1.09 (0.88-1.34)  ztvBMD  1.48 (1.15-1.91) | zcsBMD  0.99 (0.82-1.19)  ztvBMD  1.69 (1.35-2.10) | zcsBMD  1.44 (0.85-2.43)  ztvBMD  1.71 (1.00-2.92) |
| **Cox Proportional Hazards Model for outcome hip fracture (HR (95% CI))** | | | | |
|  | **Combined** (n=4904, Events = 130) | **Normal**  (n=2375, Events =18) | **Osteopenia**  (n=2202, Events=80) | **Osteoporosis**  (n=327, Events = 32) |
| **Model 1** | 2.60 (1.99-3.38) | 2.51 (0.81-7.75) | 3.78 (1.98-7.23) | 3.29 (1.35-8.01) |
| **Model 2** | 1.92 (1.52-2.41) | 1.61 (0.86-3.01) | 1.51 (1.03-2.23) | 1.74(0.26-3.91) |
| **Model 3** | 2.49 (1.93-3.21) | 2.33 (1.02-5.36) | 2.80 (1.75-4.48) | 1.38 (0.65-2.94) |
| **Model 4** | zcsBMD: 1.26 (0.96-1.66)  ztvBMD: 2.16 (1.59-2.93) | zcsBMD  1.29 (0.64-2.61)  ztvBMD  2.11 (0.86-5.19) | zcsBMD: 1.29 (0.88-1.89) ztvBMD: 2.64 (1.64-4.27 | zcsBMD: 1.36 (0.62-2.95) ztvBMD: 1.77 (0.78-3.98) |
| Model 1: Cohort + Age + Sex + Height + Weight + Smoking Status + Systemic corticosteroid use + Alcohol use + zaBMD  Model 2: Cohort + Age + Sex + Height + Weight + Smoking Status + Systemic corticosteroid use + Alcohol use + zcsBMD  Model 3: Cohort + Age + Sex + Height + Weight + Smoking Status + Systemic corticosteroid use + Alcohol use + ztvBMD  Model 4: Cohort + Age + Sex + Height + Weight + Smoking Status + Systemic corticosteroid use + Alcohol use + zcsBMD + ztvBMD  zaBMD – Z-score areal bone mineral density, zcsBMD – Z-score cortical surface bone mineral density, ztvBMD – Z-score trabecular volumetric bone mineral density | | | | |
