# Supplementary material for: Use of DXA-derived 3D-modeling, as implemented by 3D-Shaper, for the assessment of fracture risk in a population-based setting
Source: J Bone Miner Res. 2025 Sep 2;41(2):128–35. doi: 10.1093/jbmr/zjaf120 (PMC12865847; doi:10.1093/jbmr/zjaf120)
Supplement: R1_Supplementary_Table_6_zjaf1 [file r1_supplementary_table_6_zjaf1.docx]

**Supplementary Table 6.** Comparative analysis of baseline characteristics between individuals with discordant values of aBMD, csBMD and tvBMD at the neck, trochanter or total hip.

|  | **Combined**  **(n=4855)** | **Femoral Neck (n=53)** | **p-value** | **Combined (n=4861)** | **Trochanter (n=47)** | **p-value** | **Combined (N=4873)** | **Total hip (n=35)** | **p-value** |
| --- | --- | --- | --- | --- | --- | --- | --- | --- | --- |
| **Age,** *yrs*(SD) (mean) | 67.4 (10.1) | 65.5(11.6) | 0.14 | 67.4  (10.1) | 64.6 (11.3) | 0.09 | 67.5(10.1) | 61.1(11.3) | 0.002 |
| **Sex, women (n, %)** | 2705(55.7) | 32 (60.3) | 0.59 | 2708(55.7) | 29 (61.7) | 0.50 | 2713 (55.7) | 24 (69) | 0.17 |
| **Height,** cms (SD) mean | 168.4 (9.4) | 168.8 (10.2) | 0.81 | 168.5(9.4) | 166.3 (10.9) | 0.18 | 168.4(9.4) | 166.4(10.7) | 0.28 |
| **Weight,** *kgs* (SD) | 78.2 (14.0) | 77.5(16.5) | 0.74 | 78.2(14.0) | 78.7 (16.4) | 0.83 | 78.2(14.0) | 76.7(16.8) | 0.60 |
| **BMI** (SD) | 27.5 (4.2) | 27.1 (4.9) | 0.54 | 27.5(4.2) | 28.4(4.6) | 0.20 | 27.5(4.1) | 27.7(5.9) | 0.85 |
| **Alcohol,** *g/p/d* (SD) | 10.9 (13.1) | 9.0 (10.7) | 0.21 | 10.9(13.1) | 9.4(11.4) | 0.37 | 10.9(13.1) | 6.4(7.2) | 0.0007 |
| **Smoking (n, %)**  Never  Past  current | 1489 (30.7)  2591 (53.4)  775 (15.9) | 11 (20.8)  28(52.8)  14(26.4) | 0.08  (F) | 1486(30.6)  2596(53.4)  779(16.0) | 14 (29.8)  23(48.9)  10(21.3) | 0.61 | 1487 (30.5)  2602 (53.4)  784(16.1) | 13 (37.1)  17(48.6)  5 (14.3) | 0.68 |
| **Systemic corticosteroid medication (*n*, %)** | 57 (1.2) | 0(0) | < 0.001 | 56(1.2) | 1(2.0) | 0.42 | 56 (1.0) | 1(2.9) | 0.34 |
| **Cohort(n, %)**  I  II  III | 2333(48.1)  913 (18.8)  1609 (33.1) | 25(47.2)  8 (15.1)  20(57.7) | 0.09 | 2334(48.1)  916(18.8)  1611(33.1) | 19(40.4)  5(10.6)  23(49.0) | 0.15 | 2346(48.1  916(18.8)  1611(33.1) | 7(0.2)  5(14.3)  23(65.7) | 0.0002 |
| **Diag (n, %) (F)**  Healthy  Osteopenia  Osteoporosis | 2338(48.2)  2196(45.2)  321(6.6) | 37(69.8)  10(18.9)  6(11.3) | 0.0002 | 2345(48.2)  2191(45.1)  32(6.7) | 30(63.8)  15(31.9)  2(4.3) | 0.21 | 2351(48.2)  2197(45.1)  325(6.7) | 24(68.6)  9(25.7)  2(5.7) | 0.044 |
| **Any-type Fracture (n, %)** | | | | | | | | | |
| No | 3843 (79.2) | 46(86.8) |  | 3850(79.2) | 39(83.0) |  | 3863(79.3) | 26(74.3) |  |
| Yes | 1012(20.8) | 7(13.2) | 0.23 | 1011(20.8) | 8(17.0) | 0.65 | 1010(20.7) | 9(25.7) | 0.61 |
| **Hip Fracture (n, %) (F)** | | | | | | | | | |
| No | 4685 (96.5) | 52(98.1) |  | 4691(96.5) | 46 (98.0) |  | 4703(96.5) | 34 (97.1) |  |
| Yes | 170 (3.5) | 1(1.9) | 1 | 170(3.4) | 1(2.0) | 0.91 | 170 (3.5) | 1 (2.9) | 0.81 |
| **aBMD,** *g/cm2* (SD) | 0.90(0.14) | 0.98(0.22) | 0.01 | 0.79(0.16) | 0.90(0.25) | 0.004 | 0.95(0.16) | 1.07(0.23) | 0.002 |
| **csBMD,** *mg/cm2* (SD) | 124.1(21.3) | 153.7(38.9) | 0.002 | 149.7(26.2) | 166.3(38.3) | <0.0001 | 161.4(25.5) | 184.7(34.0) | 0.0002 |
| **tvBMD,** *mg/cm3* (SD) | 195.9(50.5) | 240.1(98.4) | 0.0008 | 136.1(39.9) | 167.1(73.5) | <0.0001 | 157.4(40.8) | 208.9(76.3) | 0.0003 |
|  | N=3375 | N=33 |  | N=3372 | N=36 |  | N=3383 | N=25 |  |
| **OADiag(%) (F)**** | 190(3.9) | 4(7.5) | 0.115 | 192(3.9) | 2(2.0) | 1 | 193(4.0) | 1(2.9) | 1 |
| ** BMI – Body Mass Index, Diag – Healthy, Osteopenia or Osteoporosis diagnosis, aBMD – areal bone mineral density, csBMD – Cortical surface bone mineral density, tvBMD – Trabecular volumetric bone mineral density, OA Diag – Osteoarthritis Diagnosis, F – Fisher analysis was performed | | | | | | | | | |
